# Anti-Biofilm and Anti-Hemolysis Activities of 10-Hydroxy-2-decenoic Acid against *Staphylococcus aureus*

**DOI:** 10.3390/molecules27051485

**Published:** 2022-02-22

**Authors:** Kuankuan Gao, Bei Su, Jing Dai, Piwu Li, Ruiming Wang, Xiaohui Yang

**Affiliations:** 1 State Key Laboratory of Biobased Material and Green Papermaking (LBMP), Qilu University of Technology (Shandong Academy of Sciences), Jinan 250353, China; 13969083326@163.com (K.G.); subei970211@163.com (B.S.); piwuli@126.com (P.L.); ruiming3k@163.com (R.W.); 2 Key Laboratory of Shandong Microbial Engineering, Qilu University of Technology (Shandong Academy of Sciences), Jinan 250353, China; jingdai_flower@sina.com

**Keywords:** 10-hydroxy-2-decenoic acid, *Staphylococcus aureus*, biofilm, hemolytic activity

## Abstract

Persistent infections caused by *Staphylococcus aureus* biofilms pose a major threat to global public health. 10-Hydroxy-2-decenoic acid (10-HDA), a main fatty acid in royal jelly, has been shown to possess various biological activities. The purpose of this study was to explore the effects of 10-HDA on the biofilms and virulence of *S. aureus* and its potential molecular mechanism. Quantitative crystal violet staining indicated that 10-HDA significantly reduced the biofilm biomass at sub-minimum inhibitory concentration (MIC) levels (1/32MIC to 1/2MIC). Scanning electron microscope (SEM) observations demonstrated that 10-HDA inhibited the secretion of extracellular polymeric substances, decreased bacterial adhesion and aggregation, and disrupted biofilm architecture. Moreover, 10-HDA could significantly decrease the biofilm viability and effectively eradicated the mature biofilms. It was also found that the hemolytic activity of *S. aureus* was significantly inhibited by 10-HDA. qRT-PCR analyses revealed that the expressions of global regulators *sarA*, *agrA*, and α-hemolysin gene *hla* were downregulated by 10-HDA. These results indicate that 10-HDA could be used as a potential natural antimicrobial agent to control the biofilm formation and virulence of *S. aureus*.

## 1. Introduction

*Staphylococcus aureus* is a pathogenic microorganism responsible for a variety of infectious diseases in humans, ranging from skin infection to severe tissue infection and bacteremia [1,2]. It is also one of the foodborne pathogens that causes food contamination and food poisoning [3,4]. Planktonic *S. aureus* has a strong ability to form biofilms on the surfaces of medical devices and food equipment [5,6,7]. Biofilms are complex microbial ecosystems formed by bacterial communities embedded in a self-produced matrix of extracellular polymeric substances (EPS), which increase bacterial resistance to antimicrobial agents and extreme environments, and promote cross-contamination [8,9,10]. Thus, the bacteria in biofilms are more difficult to eradicate. In addition, *S. aureus* can produce several virulence factors, such as hemolysin, enterotoxin, and coagulase, which are associated with the pathogenicity of *S. aureus* [11]. In particular, α-hemolysin is an important virulence factor involved in the pathogeneses of skin infections, pneumonia, and sepsis [12]. In the past few years, numerous antibiotics and disinfectants have been used to combat pathogens. However, the long-term use of antibiotics and synthetic chemicals leads to the emergence of resistance, high toxicity, and other negative effects on human health and the environment [13,14]. Therefore, it is necessary to explore novel and safe antibacterial agents. Currently, natural products are considered to be an important source of “eco-friendly” antimicrobials [14]. Natural products from plants, animals, and microorganisms have been widely discovered, providing attractive candidates for the development of antibacterial agents [15,16,17,18]. 

10-Hydroxy-2-decenoic acid (10-HDA), also known as royal jelly acid, is the major fatty acid component of royal jelly. 10-HDA has been reported to possess a variety of activities beneficial to human health, including anti-inflammatory [19,20], antitumor [21,22,23], immunomodulatory [24,25], and lifespan-extending activities [26]. We have previously reported that 10-HDA has an antibacterial activity against planktonic *S. aureus* by destroying the integrity of the cell membrane [27]. However, the inhibition effects of 10-HDA on *S. aureus* biofilms and virulence production remain unclear. Thus, the effects of 10-HDA on biofilm formation and the mature biofilms of *S. aureus* were investigated in this study by detecting the biomass, morphology, cell viability, and extracellular polymeric substances (EPS) production of biofilms. The effect of 10-HDA on the hemolytic activity of *S. aureus* was also evaluated. Furthermore, the molecular mechanisms of the 10-HDA-mediated inhibition of biofilm formation and virulence production were explored through qRT-PCR analysis. 

## 2. Results and Discussion

### 2.1. Effect of 10-HDA on the Growth of Planktonic Bacteria and Biofilm Biomass

**Figure 1:**
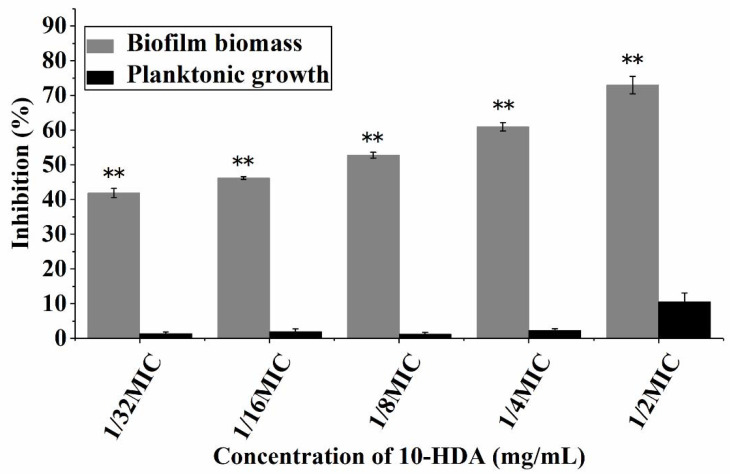
Effect of 10-HDA at sub-MIC levels on the planktonic growth and biofilm biomass of *S. aureus*. ** *p* < 0.01.

The minimum inhibitory concentration (MIC) of 10-HDA against *S. aureus* ATCC25923 was 2.25 mg/mL. The influence of 10-HDA at sub-MIC levels on the planktonic growth and biofilm formation of *S. aureus* is shown in Figure 1. The planktonic growth of *S. aureus* was not significantly inhibited after 10-HDA treatment at concentrations ranging from 1/32MIC (0.07 mg/mL) to 1/2MIC (1.13 mg/mL). However, 10-HDA significantly reduced the biomass of *S. aureus* biofilms in a dose-dependent manner (*p* < 0.01). When treated with 10-HDA at concentrations from 1/32MIC to 1/2MIC, the biofilm biomass was reduced by 41.9% to 72.9%. The results indicated that the inhibitory effect of 10-HDA on *S. aureus* biofilm formation was attributed to the anti-biofilm ability of 10-HDA rather than the inhibition of the bacterial growth, and this feature may prevent the emergence of bacterial resistance [28].

### 2.2. Effect of 10-HDA on the Morphology of S. aureus Biofilms

**Figure 2:**
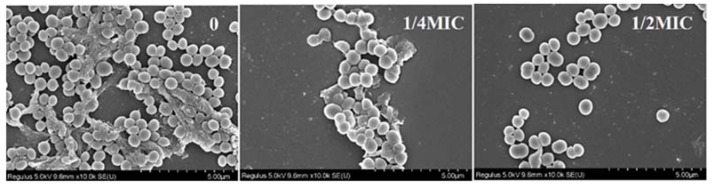
SEM images (×10,000) of *S. aureus* biofilms with 10-HDA treatment at the indicated concentrations.

The morphological and microstructural changes in *S. aureus* biofilms treated with 10-HDA were observed by SEM (Figure 2). The biofilms without 10-HDA treatment were densely packed with bacteria, and the bacteria were enveloped in the EPS structure of the biofilm. In contrast, after 10-HDA treatment at 1/4MIC and 1/2MIC, the EPS structure almost disappeared in the microscopic field, and the biofilms exhibited fewer cells that were loosely attached to the glass slides. In order to form biofilms, bacteria produce EPS to mediate their adhesion to inert surfaces and promote their survival in extreme environments [9,29]. In this study, SEM observations demonstrated that 10-HDA inhibited EPS production, decreased bacterial adhesion and aggregation, and disrupted biofilm architecture, which conferred the remarkable inhibition effect of 10-HDA on *S. aureus* biofilm formation. 

### 2.3. Effect of 10-HDA on Cell Viability during Biofilm Formation

**Figure 3:**
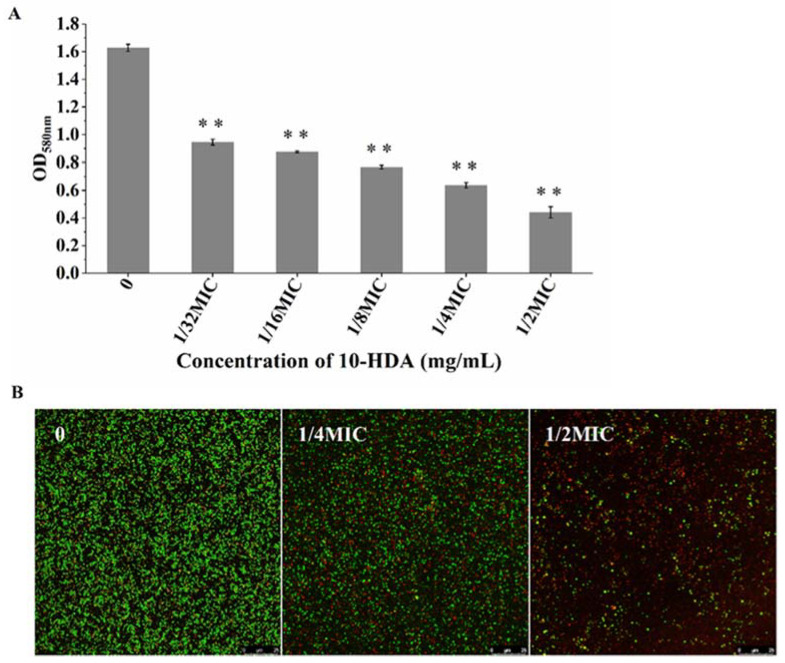
Effect of 10-HDA on bacterial viability during biofilm formation. (**A**) Effect of 10-HDA on the metabolic activity of the cells in *S. aureus* biofilms, as analyzed by MTT assay. ** *p* < 0.01. (**B**) CLSM images of *S. aureus* biofilms. Green fluorescence: live cells; red fluorescence: dead cells.

The effect of 10-HDA on the metabolic activity of *S. aureus* biofilms was measured by MTT assay. As shown in Figure 3A, the metabolic activity of cells in the biofilms significantly decreased upon treatment with 10-HDA from 1/32MIC to 1/2MIC. After treatment with 1/2MIC of 10-HDA, the metabolic activity of the biofilm cells decreased by 73.6%.

Moreover, the effect of 10-HDA on bacterial viability during biofilm formation was investigated by Confocal Laser Scanning Microscopy (CLSM). SYTO 9 dye can stain live bacteria with intact membranes and fluoresce green, whereas PI dye can stain dead bacteria with damaged membranes and fluoresce red [30]. The CLSM images are shown in Figure 3B. The biofilms without 10-HDA treatment presented a dense green fluorescence, indicating that the bacteria were alive. Meanwhile, in the treatment with 10-HDA at 1/4MIC, the green fluorescence presented by the biofilms decreased obviously, and the red fluorescence increased compared with the control. After the treatment with 10-HDA reached 1/2MIC, most of the cells appeared red in the field of vision. These images, as well as the MTT results, indicate that 10-HDA had a significant inhibitory effect on the cell viability of *S. aureus* during biofilm formation. Similar observations were reported for other antibiofilm agents, such as gallic acid and shikimic acid, which inhibited biofilm formation and reduced the cellular viability of bacteria, suggesting that these agents might destroy the EPS structure and enter biofilms to inactivate the protected cells [31,32,33].

### 2.4. Effect of 10-HDA on EPS Production

**Figure 4:**
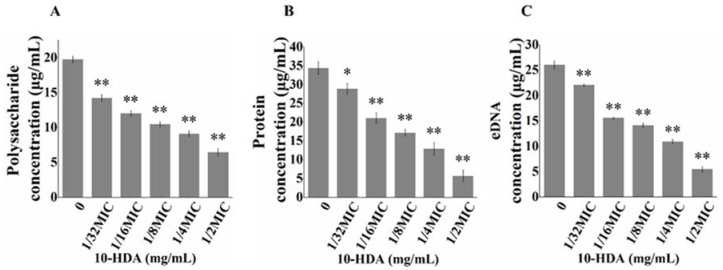
The contents of extracellular (**A**) polysaccharides, (**B**) proteins, and (**C**) eDNA in *S. aureus* biofilms after 10-HDA treatment. * *p* < 0.05, ** *p* < 0.01.

EPS are biopolymers produced by bacteria, mainly composed of extracellular polysaccharides, proteins, and extracellular DNA (eDNA). They provide a stable architecture for biofilms to mediate bacterial adhesion and aggregation [9]. Thus, the destruction of EPS is also an important indicator for evaluating anti-biofilm activity. As shown in Figure 4A–C, the contents of extracellular polysaccharides, proteins, and eDNA in the biofilms decreased significantly with the increase of the 10-HDA concentration. When the 10-HDA concentration was 1/2MIC, the inhibition rate of extracellular polysaccharides, proteins, and eDNA reached 67.4%, 83.5%, and 76.8%, respectively. This result suggested that 10-HDA could reduce EPS production to prevent the formation of biofilms, which was consistent with the results of the SEM images above.

### 2.5. Effect of 10-HDA on Mature Biofilms

**Figure 5:**
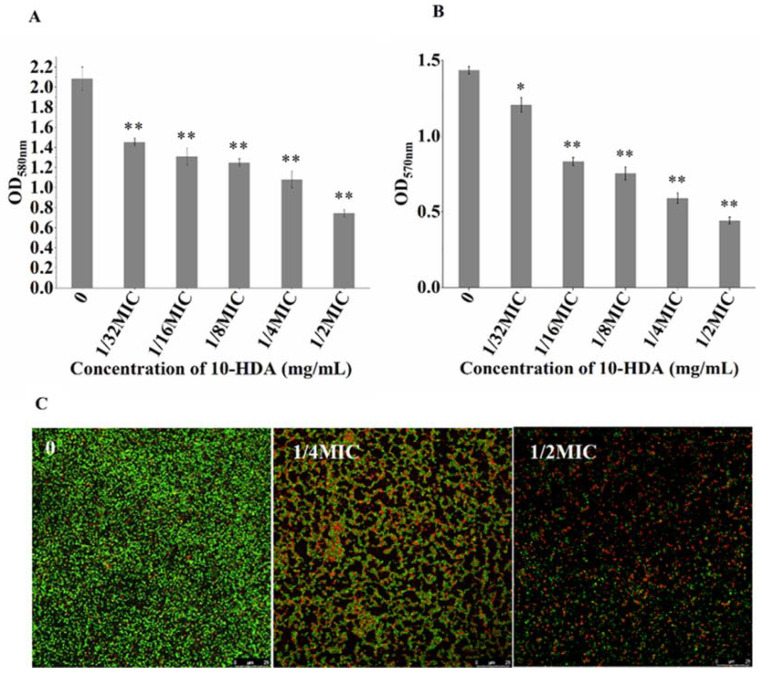
(**A**) Effect of 10-HDA on *S. aureus* mature biofilms, as analyzed by crystal violet staining method. (**B**) MTT assay on the effect of 10-HDA on the cell metabolic activity in *S. aureus* mature biofilms. * *p* < 0.05, ** *p* < 0.01. (**C**) CLSM images of *S. aureus* mature biofilms. Green fluorescence: live cells; red fluorescence: dead cells.

Mature biofilms are considered to be highly organized ecosystems with many water channels for material exchange [34]. Thus, mature biofilms possess a higher viability and are extremely difficult to eradicate. Our results showed that 10-HDA significantly reduced the biomass of mature biofilms at concentrations ranging from 1/32MIC to 1/2MIC (*p* < 0.01). When treated with 10-HDA at 1/2MIC, the biofilm mass decreased by 64.3% (Figure 5A). Moreover, the MTT assay indicated a substantial reduction (15.9% to 69.2%) of the cell metabolic activity in the *S. aureus* mature biofilms after treatment with 10-HDA from 1/32MIC to 1/2MIC compared with the control (Figure 5B). CLSM images further confirmed the inhibition of the cell viability of mature biofilms by 10-HDA. With the increase in the 10-HDA concentration, the green fluorescence was gradually reduced and more red fluorescence appeared in the field of vision (Figure 5C). Collectively, these results indicate that 10-HDA could effectively eradicate the mature biofilms and reduce the bacterial viability through the mature biofilms.

### 2.6. Effect of 10-HDA on Hemolytic Activity

**Figure 6:**
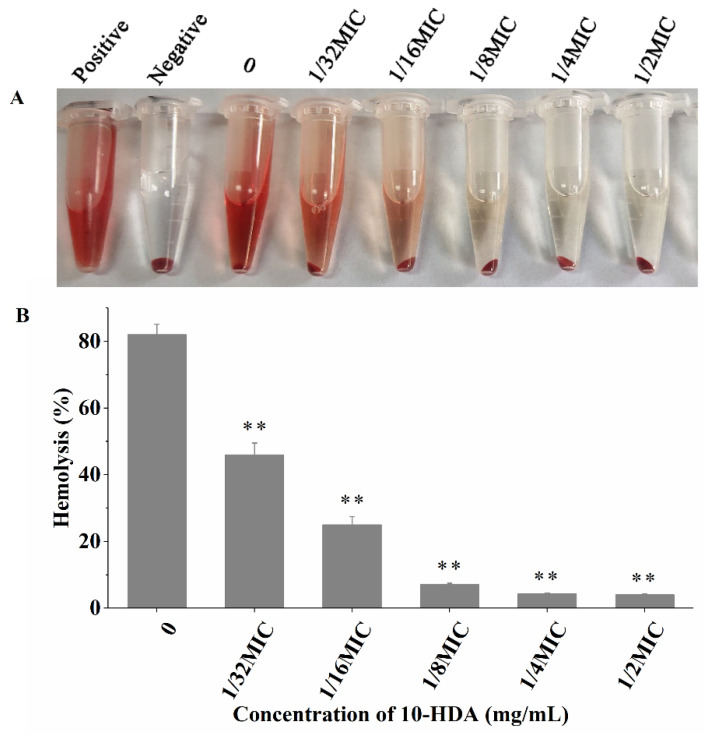
Efficacy of 10-HDA in inhibiting the hemolytic activity of *S. aureus*. (**A**) Qualitative and (**B**) quantitative analysis results are shown. ** *p* < 0.01.

Staphylococcal hemolysins are important virulence factors contributing to bacterial invasion [35]. Alpha-hemolysin is considered the main pathogenicity factor because of its hemolytic effects. It attaches to the host red blood cells and creates pores in the cell membrane to disrupt the cellular homeostasis, eventually causing cell death [36]. In this study, the results show that the hemolytic activity of *S. aureus* treated with 10-HDA was obviously reduced when compared with the control (without 10-HDA treatment). The percent of hemolysis was dramatically reduced from 82.1% (untreated control) to 46.0%, 25.0%, 7.2%, 4.2%, and 4.0% after 10-HDA treatment at 1/32MIC, 1/16MIC, 1/8MIC, 1/4MIC, and 1/2MIC, respectively (Figure 6). These results suggest that 10-HDA has a strong inhibitory effect on hemolysin production in *S. aureus*.

### 2.7. Effect of 10-HDA on the Expression of Biofilm- and Virulence-Related Genes 

**Figure 7:**
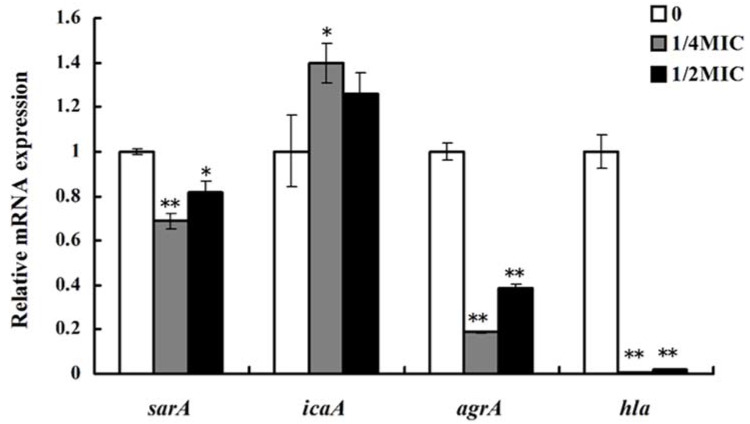
qRT-PCR results of 10-HDA’s effect on the transcription of biofilm- and virulence-related genes. * *p* < 0.05, ** *p* < 0.01.

A gene expression analysis was performed to examine the anti-biofilm and anti-hemolysis activities of 10-HDA against *S. aureus* at the transcription level. As shown in Figure 7, when treated with 10-HDA at 1/4MIC and 1/2MIC, the expressions of *sarA*, *agrA*, and *hla* were significantly downregulated, and gene *icaA* was upregulated when compared with the control.

The *sar* and *agr* loci are two important global regulators of biofilm formation and virulence production in *S. aureus*. *sarA* has been shown to be involved in biofilm development, and the *sarA* mutation decreases the ability of *S. aureus* to form biofilms [37,38,39]. As for *agr* loci, their influence on biofilm formation varies with the growth conditions, which may reflect the response of the Agr regulatory system to the external environment [40,41]. Moreover, the *agr* and *sarA* loci control toxin production in *S. aureus*. It was reported that both the *agr* and *sarA* loci affected hemolysins synthesis by exerting positive impacts on alpha-hemolysin gene *(hla)* expression [42,43,44]. We found that *sarA, agrA,* and *hla* expressions were downregulated in 10-HAD-treated samples, indicating that *sarA* and *agrA* might be key regulatory factors responsible for the inhibition of biofilm and hemolytic activity. The *ica* operon encodes the enzymes that synthesize polysaccharide intercellular adhesin for its participation in biofilm formation [32,45]. However, our results showed that the interference of 10-HDA in biofilm formation was accompanied by the upregulation of *icaA*. Some studies have shown that mutations of the *ica* locus do not reduce the ability of biofilm formation in certain *S. aureus* strains, suggesting an *ica*-independent pathway for biofilm formation [46,47,48]. Taken together, these results suggest that 10-HDA inhibits the biofilm formation and hemolytic activity of *S. aureus* by repressing the transcription of *sarA*, *agrA*, and *hla*.

## 3. Materials and Methods

### 3.1. Antimicrobial and Bacterial Strain

10-HDA (≥98%) was purchased from Shanghai Macklin Biochemical Technology Co., Ltd. (Shanghai, China). The standard strain *S. aureus* ATCC25923 was purchased from the China Center of Industrial Culture Collection (Beijing, China). 10-HDA was dissolved in dimethyl sulfoxide (DMSO) (0.5%, *v*/*v*) and serially diluted to different concentrations by Tryptic Soytone Broth (TSB) liquid medium. 

### 3.2. Effect of 10-HDA on the Planktonic Growth and Biofilm Biomass of S. aureus

*S. aureus* was cultured in TSB liquid medium for 12 h. Then, *S. aureus* suspension (1 × 10^6^ CFU/mL) was incubated with 1/32MIC (0.07 mg/mL), 1/16MIC (0.14 mg/mL), 1/8MIC (0.28 mg/mL), 1/4MIC (0.56 mg/mL), and 1/2MIC (1.13 mg/mL) of 10-HDA in a 96-well plate at 37 °C for 24 h. The sample of bacterial suspension treated with DMSO was used as the control. The bacterial growth was measured by a microplate reader (Bio-Tek, Winooski, VT, USA) at 600 nm. For the biofilm biomass analysis, the bacteria were cultured in the same way as above. After incubation, the planktonic cells were removed by washing the wells three times with PBS. The biofilms were stained with 0.5% crystal violet for 10 min. Finally, the biofilms were dissolved in 95% ethanol, and the absorbance at 580 nm was measured on a microplate reader. The percentage of inhibition of the planktonic growth or biofilm formation was calculated using the following equation [33]:

Inhibition (%) = [(Control OD_600_/_580 nm_ − Treated OD_600/580 nm_)/Control OD_600/580 nm_] × 100

### 3.3. SEM Analysis

*S. aureus* suspension (1 × 10^6^ CFU/mL) was incubated with 1/4MIC and 1/2MIC of 10-HDA on glass slides (φ 14 mm) in a 24-well plate for 24 h at 37 °C. The samples of bacterial suspension treated with DMSO were used as the control. After incubation, the glass slides were then washed with sterile PBS, and 2.5% (*v*/*v*) glutaraldehyde was used to fix the biofilms for 6 h at 4 °C. Subsequently, the biofilms were dehydrated for 10 min using an ethanol gradient (25%, 50%, 75%, 90%, and 100%). Finally, the samples were coated with gold and observed under SEM (Regulus8220, Hitachi, Tokyo, Japan).

### 3.4. MTT Assay

*S. aureus* biofilms with 10-HDA treatment at concentrations ranging from 1/32MIC to 1/2MIC were prepared according to the method in Section 3.2. The samples of bacterial suspension treated with DMSO were used as the control. After removing the planktonic cells, the cells in the biofilms were incubated with MTT (5 mg/mL) for 2 h. During incubation, the dehydrogenase system of active cells can reduce MTT to formazan crystals. Subsequently, the MTT solution was aspirated, and the formazan solubilization solution was added to the wells to dissolve the formazan crystals in the cells. The cell viability was proportional to the concentration of formazan, which could be detected by a microplate reader at 570 nm [49]. 

### 3.5. Confocal Laser Scanning Microscopy (CLSM) Observation

*S. aureus* biofilms with 10-HDA treatment were prepared in a 24-well plate according to the method in Section 3.3. After removing the planktonic cells, the biofilms were then stained with dyes (SYTO 9 and PI) according to the instructions of the LIVE/DEAD BacLight bacterial viability kit. Staining took place in the dark at room temperature for 15 min. The biofilms were then washed with sterile water and observed under CLSM (SP8, Leica, Wetzlar, Germany).

### 3.6. Determination of Extracellular Polymeric Substances Contents

*S. aureus* biofilms with 10-HDA treatment were prepared on glass slides (φ 14 mm) in a 24-well plate. After removing the planktonic cells, the samples were sonicated with PBS and centrifuged for 20 min at 6000 r/min. The supernatant was collected and used to measure the contents of extracellular polysaccharide, protein, and DNA. The extracellular polysaccharide content was measured by the anthrone-sulfuric acid method [50], and the extracellular protein was measured using a BCA Protein Assay Kit (Sangon, Shanghai, China). The extracellular DNA (eDNA) was extracted by the phenol-chloroform method, and the absorbance was recorded at 260 nm.

### 3.7. Effect of 10-HDA on Mature Biofilms

*S. aureus* suspension (1 × 10^6^ CFU/mL) without 10-HDA was cultured in 96-well or 24-well microplates at 37 °C for 48 h to obtain the mature biofilms. After removing the planktonic cells, the biofilms were treated with different concentrations of 10-HDA at 37 °C for 24 h. The samples were analyzed for biofilm biomass and cell viability as described above.

### 3.8. Measurement of Hemolytic Activity

*S. aureus* suspension was incubated with different concentrations of 10-HDA in a centrifuge tube for 24 h at 37 °C. After centrifugation (12,000 rpm, 5 min), 100 μL of the supernatant was taken from each tube and incubated with 25 μL of freshly washed sheep red blood cells in 875 μL of PBS at 37 °C for 1 h. The incubation of Triton X-100 and sheep red blood cells was used as the positive control, and the incubation of PBS and sheep red blood cells served as the negative control. After centrifugation (3000 rpm, 5 min), the absorbance of supernatants at 450 nm was examined. The percentage of hemolysis value was calculated by comparing it with the positive control (100% hemolysis) [51].

### 3.9. qRT-PCR

qRT-PCR was applied to analyze the effects of 10-HDA on the transcription of genes associated with biofilm development and virulence. The gene-specific primers are listed in Table 1, and the 16s rRNA housekeeping gene was used as an internal standard. Briefly, *S. aureus* suspension (1 × 10^6^ CFU/mL) with 0, 1/4MIC, and 1/2MIC of 10-HDA was incubated at 37 °C for 24 h. Then, the biofilms were collected, and total RNA was isolated using the Bacteria Total RNA Isolation Kit (Sangon Biotech, Shanghai, China). qRT-PCR was performed using SYBR Green qPCR Mix on a real-time PCR system. Data were analyzed by the 2^−^^△△CT^ method.

### 3.10. Statistical Analysis

All experiments were performed in triplicate, and the data were presented as the mean ± SD. A significant difference between groups was determined by a one-way analysis of variance using IBM SPSS 22.0 (IBM, Armonk, NY, USA). 

## 4. Conclusions

The effects of 10-HDA on the biofilms and virulence of *S. aureus* were evaluated for the first time. Sub-MICs of 10-HDA could effectively inhibit the formation of biofilms and eradicate the mature biofilms of *S. aureus*, as confirmed by significant reductions in biofilm biomass, cell viability, and EPS production. Moreover, the biofilm structure was visibly damaged after 10-HDA treatment. In addition, 10-HDA has a strong inhibitory effect on hemolysin production in *S. aureus*. qRT-PCR results indicate that 10-HDA prevents biofilm formation and hemolytic activity by repressing the transcription of the global regulators *sarA*, *agrA*, and α-hemolysin gene *hla*. The findings reveal that 10-HDA has remarkable inhibition effects on the biofilm formation and virulence of *S. aureus*, supporting its use as a potential natural antimicrobial agent to combat *S. aureus*.

## Figures and Tables

**Table 1:** Primer sequences for quantitative RT-PCR.

Gene	Primer
*sarA*	Forward 5’-CATCAGCGAAAACAAAGAGAAA-3’
	Reverse 5’-TGTTTGCTTCAGTGATTCGTTT-3’
*icaA*	Forward 5′-TTTCGGGTGTCTTCACTCTAT-3′
	Reverse 5′-CGTAGTAATACTTCGTGTCCC-3′
*agrA*	Forward 5′-CAACTCGCTGACCACCTAC-3′
	Reverse 5′-TGGAGAGAGAAACCGTGC-3′
*hla*	Forward 5′-TTGGTGCAAATGTTTC-3′
	Reverse 5′-TCACTTTCCAGCCTACT-3′
*16s rRNA*	Forward 5′-ACTGGGATAACTTCGGGAAA-3′
	Reverse 5′-CGTTGCCTTGGTAAGCC-3′

## Data Availability

Not applicable.

## Sample Availability

The samples are available from the authors.
